# First person – Subhradip Das

**DOI:** 10.1242/bio.062174

**Published:** 2025-08-04

## Abstract

First Person is a series of interviews with the first authors of a selection of papers published in Biology Open, helping researchers promote themselves alongside their papers. Subhradip Das is first author on ‘ Caspar modulates primordial germ cell fate both in Oskar-dependent and Oskar-independent manner’, published in BiO. Subhradip conducted the research described in this article while a PhD scholar in Dr Girish Ratnaparkhi's lab at the Indian Institute of Science Education and Research, Pune, India. He is now a deputy research scientist in the lab of Dr Avijit Ghosh at GCC Biotech, Kolkata, India, investigating germline stem cells and post-translational modifications in early embryonic development.


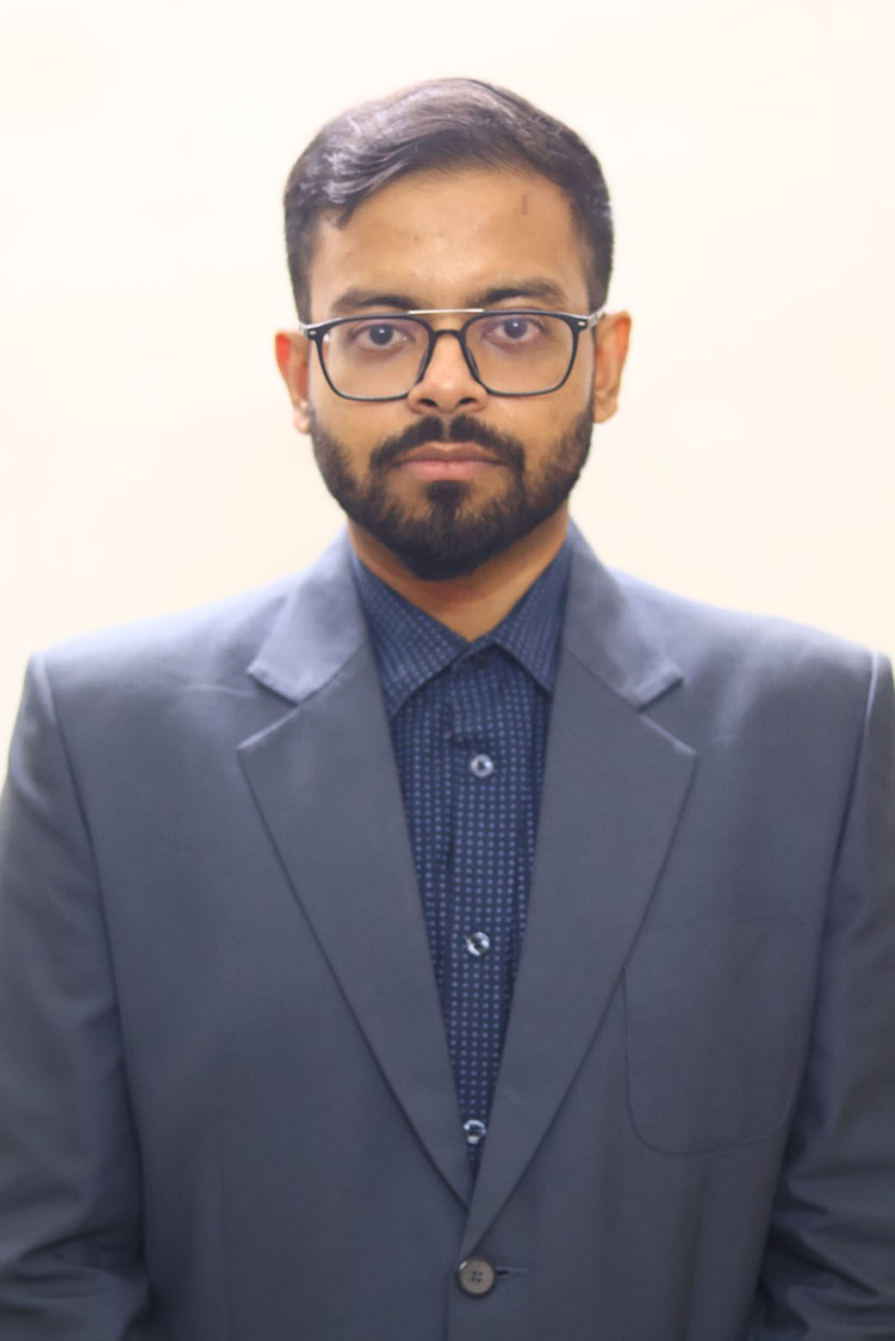


**Subhradip Das**

**Describe your scientific journey and your current research focus**

My interest in science took root during my higher secondary education, where the intricacies of biology first sparked my curiosity. This passion led me to pursue a BSc and MSc in biotechnology, during which I became deeply fascinated by the elegance and vast potential of the subject. I began my doctoral studies at IISER Pune under the guidance of Dr Girish Ratnaparkhi, where I initially explored the role of SUMOylation in early developmental proteins using *Drosophila melanogaster* as a model organism. As my research progressed, I developed a strong interest in germline stem cell biology. My PhD work focused on a protein called Caspar, investigating its role in germline cell fate specification. Currently, I am working as a deputy research scientist in the molecular biology department at GCC Biotech. My primary focus is on the development, modification, and optimization of diagnostic kits and molecular assays. Additionally, I am involved in formulating and refining research reagents and kits tailored for academic laboratories. Through this journey, my aim has been to bridge fundamental research with practical applications, contributing both to scientific understanding and technological innovation.

**Who or what inspired you to become a scientist?**

My father, a teacher by profession, has been the most consistent source of inspiration in my life. He always encouraged me to pursue my curiosity and to never stop asking questions. His belief in the power of knowledge instilled in me a lifelong love for learning. I was drawn to biology during my college years. The intricate architecture of the human body – so precise, yet so mysterious – captivated me. Biology, to me, is more than a subject; it is the language of life. The idea that we can understand, manipulate, and even rewrite the genetic code – the fundamental blueprint of life – felt like holding a divine tool. It is the power to heal, to correct nature's flaws, and sometimes, to step into the shoes of the creator. I have also found great inspiration in the lives and work of legendary scientists. The fearless curiosity of Rosalind Franklin, whose X-ray diffraction images led to the discovery of the DNA double helix; the brilliance of Gregor Mendel, who laid the foundations of genetics; and the transformative vision of Jennifer Doudna, whose work with CRISPR has revolutionised gene editing – all of them showed me that science is both an art and a revolution. Their contributions remind me that science isn't just about discovery – it's about daring to ask the impossible and then trying to make it real. Their stories, combined with my personal journey, fuel my desire to contribute meaningfully to the ever-evolving world of biology.

**How would you explain the main finding of your paper?**

During early development, an embryo sets aside a small number of cells that will later become eggs or sperm – these are called germ cells. Our research focused on a protein called Caspar, and we found that it plays a key role in controlling how many germ cells are formed and whether they develop correctly. Caspar influences this process in two important ways. First, it helps regulate another key protein that's essential for germ cell formation. Second, it affects the movement and behaviour of structures inside the cell, like centrosomes, which are critical for dividing and organizing cells during development. Without Caspar, germ cells are often fewer in number, misplaced, or unable to develop properly. Overall, our study reveals that Caspar is a dual-function regulator; it controls both molecular signals and cellular mechanics to ensure the proper formation, number, and identity of germ cells in the developing embryo.

**What are the potential implications of this finding for your field of research?**

These studies reveal that a protein called Caspar plays a central role in the early development of future reproductive cells in fruit fly embryos. What's fascinating is that this protein was previously known for its role in immune defence, but here it shows up as a crucial organiser in the formation of germ cells – the cells that eventually give rise to eggs or sperm. This dual role suggests that nature reuses the same tools for vastly different jobs, depending on the context. The discovery deepens our understanding of how early embryos carefully balance and distribute the necessary materials to set aside the future germline from the rest of the body. By regulating both the storage and movement of certain key elements, Caspar ensures these germ cells form in the right number and location. This finding could ripple outward, offering insights into how similar mechanisms might work in other organisms, including humans. It also touches upon a philosophical truth in biology: the same molecules that protect life may also quietly shape its beginnings.

In science, as in life, the path walked together often means more than the destination reached

**Figure:**
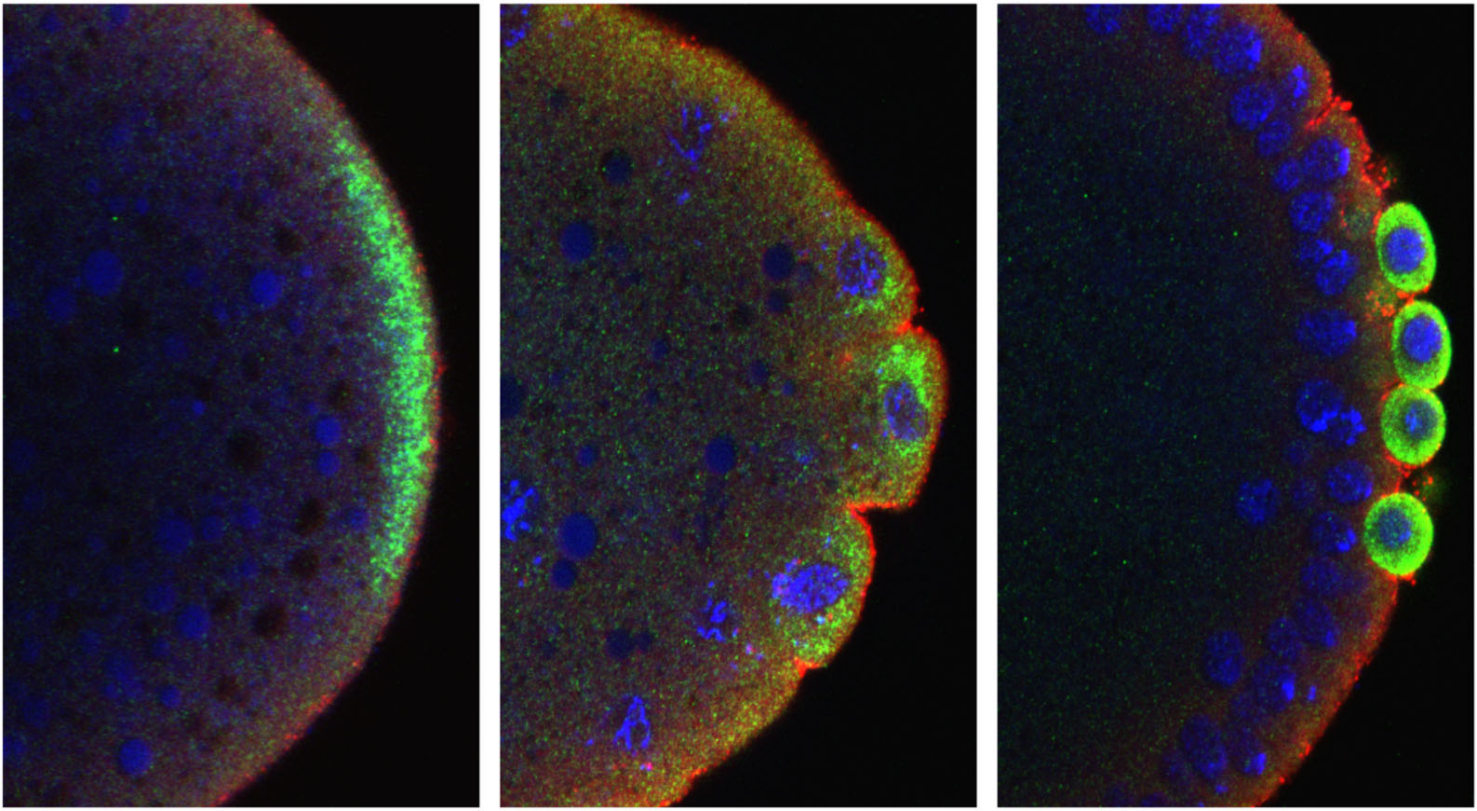
Caspar modulates primordial germ cell fate.

**Which part of this research project was the most rewarding?**

The most rewarding part of this research journey was not just the discovery itself – that a protein known for immune regulation also influences the fate of germ cells – but the collaborative spirit that emerged as the project unfolded. What began as a curious finding grew into a collective effort, enriched by the contributions of new team members, each bringing fresh energy and insight. The countless hours of discussion, troubleshooting experiments, and celebrating small victories together created a deep sense of shared purpose. The striking images we captured during the study added a visual poetry to the science, making the invisible visible. Working alongside Girish Deshpande, whose sharp intellect and intuitive grasp of developmental biology was both inspiring and humbling, was a highlight in itself. More than the end results, it was the journey; the learning, the failures, the resilience, which made this project unforgettable. In science, as in life, the path walked together often means more than the destination reached.

**What do you enjoy most about being an early-career researcher?**

What I cherish most about being an early-career researcher is the boundless sense of possibility. Every day offers a new question, a fresh puzzle, and the chance to glimpse something the world has never seen before. It's both humbling and exhilarating to know that even a small discovery can illuminate a corner of biology and echo far beyond the lab. My love for biology runs deep, it's not just a profession, but a passion that fuels my curiosity and creativity. To explore the unknown, to follow ideas with wonder, and to contribute meaningfully to both science and society feels incredibly fulfilling. There's a quiet joy in knowing that through this work, I am part of something larger, an ever-evolving story of life, growth, and understanding.

Science moves forward on trust

**What piece of advice would you give to the next generation of researchers?**

To the next generation of researchers, I would say this: always follow your passion, it's the compass that will guide you through the highs and lows of this journey. Research is as much about failure as it is about discovery. Learn from setbacks; they often teach you more than success ever could. A PhD is not just an academic pursuit, it's a profound lesson in patience, resilience, and integrity. Hold onto your ethics tightly. Never compromise on honesty and always acknowledge the contributions of others. Science moves forward on trust. And above all, enjoy the process. The joy of asking questions, the thrill of small breakthroughs, the beauty of working with like-minded dreamers, that's the real reward. Let the journey shape you as much as the destination.

**What's next for you?**

Next, I hope to continue pursuing my passion for research, diving deeper into the questions that fascinate me. I aim to take my work closer to the translational space where fundamental discoveries can begin to touch lives, solve real-world problems, and contribute meaningfully to society. It's not just about understanding biology but about using that understanding to make a difference. The goal is to bridge the gap between the lab bench and the world outside, and to be part of science that not only explores, but also heals, helps, and inspires.
